# A Study on the Mechanism of Cinobufagin in the Treatment of Paw Cancer Pain by Modulating Local **β**-Endorphin Expression *In Vivo*

**DOI:** 10.1155/2013/851256

**Published:** 2013-09-25

**Authors:** Tao Chen, Wei Hu, Haibo He, Zipeng Gong, Jing Wang, Xueqin Yu, Ting Ai, Ling Zhan

**Affiliations:** ^1^College of Medical Science, China Three Gorges University, 8 University Avenue, Yichang, Hubei 443002, China; ^2^Third-Grade Pharmacological Laboratory on Traditional Chinese Medicine Approved by State Administration of Traditional Chinese Medicine, Yichang, Hubei 443002, China; ^3^Hubei Key Laboratory of Natural Products Research and Development, China Three Gorges University, Yichang, Hubei 443002, China

## Abstract

*Background*. Cinobufagin has been widely used in the treatment of carcinoma and plays an important role in the relief of cancer pain. But the involved mechanism remains unknown. *Aim*. To investigate the changes in thermal and mechanical hyperalgesia in paw cancer pain in mice and the action mechanism of cinobufagin using a paw cancer pain model. *Methods*. 60 female mice were randomly divided into 5 groups: control group, model group, cinobufagin group, cinobufagin +NAL-M group, and morphine group; except ones in control group, mice were inoculated with H22 hepatoma cells in the right hind paw. From the 9th day after inoculation, mice were administrated drug once daily lasting for 8 days. The pain behavior was determined on the 2nd, 4th, 6th, and 8th days before and after administration. On the last day, they were sacrificed. The levels of **β**-END, CRF, and IL-1**β** were analyzed by ELISA; immunohistochemistry was performed to detect the expressions of **β**-END, POMC, and **μ**-OR in the tumor and adjacent tissue. *Results*. The thresholds of thermal pain and mechanical pain were significantly increased by cinobufagin. Moreover, the expressions of **β**-END, CRF, POMC, and **μ**-OR were significantly upregulated by cinobufagin. The analgesic effect of cinobufagin was blocked by the peripheral opioid receptor antagonist NAL-M. *Conclusions*. Cinobufagin significantly relieved cancer pain in mice and raised their pain threshold, mainly upregulating the expression levels of **β**-END and **μ**-OR in the hind paw tumor and adjacent tissue.

## 1. Introduction

Cancerous pain is one of the most common symptoms of malignant neoplasm. According to statistics, about 60%–90% of patients with late-stage malignant neoplasm have varying degrees of pain, and about 30% of patients have persistent severe pain [1]. As mentioned in the three-step therapy proposed by WHO, patients should be given nonsteroidal anti-inflammatory drugs and (or) opioid drugs according to the degree of pain. Although these treatments have proper curative effect, they also have some side effects, such as addiction, tolerance, immune suppression, and gastrointestinal reactions; furthermore some of them are not effective, leaving about 45% of cancer patients without pain control [2]. Recent findings indicate that there is a considerable curative effect related to the immune analgesia mechanism *in vivo*, in which immune cells synthesize and release opioid peptides [3–5], whose activities and expressions are positively correlated. Of these peptides, beta endorphin (*β*-END) and its precursor (proopiomelanocortin, POMC) have been identified [6]. When tumor or inflammation occurs, immune cells from the peripheral recycling system access these locations to release POMC, which can be processed by proteases to form peptide hormones and factors, such as *β*-END, adrenocorticotropic hormone (ACTH), and alpha melanocyte stimulating hormone (*α*-MSH) [7]. *β*-END, synthesized by immune cells and released to the external environment after stimulation of interleukin-1 (IL-1), corticotropin releasing factor (CRF), and other factors, combines with the opioid receptors localized on peripheral sensory nerve terminals concerned with analgesia.

Cinobufagin is the main active ingredient extracted from the full skin of the Bufo bufo gargarizans Cantor. It has actions of clearing away heat and toxin, reducing swelling, and relieving pain. Recent studies reported [8–12] that cinobufagin had been widely used in the treatment of carcinoma and played an important role in relief of cancer pain, especially in relieving pain induced by hepatocarcinoma, gastric cancer, and bone cancer with minor toxic side effects. It can be used for long-term use or combined with other analgesics in order to reinforce the duration effects. Now, the potential mechanisms underlying cancer pain relief by cinobufagin remain unclear. This severely restricts the further development and use of cinobufagin. Naloxone methiodide (NAL-M) is a selective peripheral opioid receptor antagonist.

Our previous research [13] found that cinobufagin increased mouse paw withdrawal latency (PWL) during mechanical hyperalgesia and thermal hyperalgesia. Furthermore, we determined the level of *β*-END in the neighboring tissues of the H22 hepatocellular carcinoma model, and the results showed that the level of *β*-END was significantly increased compared to model group, which indicated that the antinociceptive effect of cinobufagin is due to antagonism of increased expression of *β*-END in peripheral tissues. Wang et al. [14] reported that the analgesic effect of cinobufagin could be blocked by the peripheral opioid receptor antagonist naloxone. Hence, we hypothesized that the main mechanism in the analgesic effect of cinobufagin may be enhancement of immune function in the organism to increase the level of *β*-END and therefore activate peripheral mu-opioid receptors (*μ*-ORs) to modulate peripheral carcinoma pain.

## 2. Materials and Methods

### 2.1. Materials

Cinobufagin injection (batch number: 090623-1) was produced by Anhui Jinchan Biochemistry Corporation Co. Ltd; Morphine Hydrochloride Injection (batch  number: 091217-2) was produced by Shenyang NO 1 Pharmaceutical of Dongbei Pharmaceutical Corporation; Naloxone methiodide (NAL-M) was purchased from Sigma (USA); Hamocura (batch  number:  0808131) was produced by Jiangsu Wanbang Biochemistry Corporation Co. Ltd. The voucher specimens were deposited in the National Class III Laboratory of Pharmacology on Traditional Chinese Medicine, College of Medical Sciences, China Three Gorges University.

### 2.2. Animals

Female Kunming mice initially weighting 18 ± 2 g each were purchased from the Laboratory Animal Institute of Hubei Disease Control Center (Wuhan, China). The rights of experimental animals were ensured quantum satis ad during the experiment. Mice were housed in constant conditions at a temperature of 23 ± 3°C, humidity of 60 ± 5%, and on a 12 h light-dark cycle. They were fed *ad libitum* and conditioned in a nonstressful environment for at least 1 week prior to experiments. Experiments were performed in accordance with the Guide for the Care and Use of Laboratory Animals of China, Three Gorges University, and approved by the ethics committee. The whole laboratory procedure was carried out under the permission and surveillance of the institutional animal safety committee.

### 2.3. Apparatus

MP 200A electronic balance (Shanghai Yueping, China), 2390 series IITC Von Frey electronic pain measurement instrument (Woodland Hills, USA), PL-200 caloradiance prickle apparatus (Chengdu Taimeng, China), and DM-R multifunctional microscope and measuring system (Leica, Germany) were used.

### 2.4. Methods

#### 2.4.1. Preparation of H22 Hepatoma Cells

The H22 hepatoma cell line was serially cultured in Kunming mice for three generations, and ascites were extracted after 7 days. The sample was washed in D-Hanks solution and centrifuged twice at 800 r/min × 5 min and dyed with trypan blue to detect whether the survival rate ≥95%. The cell suspension was adjusted to 6 × 107/mL, which was placed in ice until use.

#### 2.4.2. Establishment of the Hind Paw Cancer Pain Model

Mice in the model group were subcutaneously injected with 0.1 mL 6 × 107 H22 hepatoma cells, and mice in the control group were injected with 0.1 mL normal saline (NS). The whole procedure was carried out aseptically and was completed within 1 h.

#### 2.4.3. Grouping and Administration

48 mice of 60 female Kunming mice, which were administrated tumor cells, were randomly divided into 4 groups: model group, cinobufagin group (cinobufagin, 2.5 g/kg/day, i.p.), cinobufagin + NAL-M group (cinobufagin, 2.5 g/kg/day, i.p. NAL-M, 20 mg/kg/day i.p.), and morphine group (morphine, 8 mg/kg/day, i.p.), with 12 individuals in one group; the remaining 12 normal mice which were not administrated tumor cells were selected control group. The experiment mice were administrated homologous drug, respectively; mice in the control and model groups were administered NS, respectively, once daily lasting for 8 days. The pain behavior of each mouse was determined on the 2nd, 4th, 6th, and 8th days before and after treatment, respectively. On the last day of treatment the weight and pain behavior of mice were measured, respectively, and specimens were sampled for testing.

The dose of cinobufagin in person is 10 g/60 kg, according to Meeh-Rubner equation [15]:

(1) $$\begin{array}{c} A=K\frac{W^{2/3}}{10000}, \end{array}$$

where *K* is constant, *K*_man_ = 10.6, *K*_mice_ = 9.1; *W* body weight *W*_man_ = 60 kg, *W*_mice_ = 20 g., We figure out that *A*_man_ ≈ 1.6 m^2^, *A*_mice_ = 0.007 m^2^. So the dose of cinobufagin in mice is 10 g × 0.007 m^2^ ÷ 1.6 m^2^ ÷ 0.02 kg *≈* 2.19 g/kg. We chose the 2.5 g/kg of cinobufagin as the dose in mice.

#### 2.4.4. Measurement of Thermal Hyperalgesia

Thermal hyperalgesia was measured using a radiant heat pain measurement instrument in a quiet environment (room temperature 22 ± 1°C) [16]. The mice were placed in a plexiglass cage, and the experiment was performed using an intense light beam to irradiate the center skin of the right hind paw when the mice were adapted to the quiet environment, and the time taken for mice to draw back their paw was recorded. Radiant heat intensity was set to 5–15 seconds for normal mouse paw withdrawal latency (PWL). Each mouse was measured 3 times, the interval between each was 10 minutes, and the average value was calculated. An upper limit of 20 seconds was set as the PWL to prevent burns.

#### 2.4.5. Measurement of Mechanical Hyperalgesia

Mechanical hyperalgesia was measured by IITC Von Frey 2390 in a quiet environment (room temperature 22 ± 1°C) [17, 18]. The mice were placed on a special glass grid, adapted to the quiet environment, and the experiment was performed. Briefly, the center skin of the right hind paw was stimulated, and the PWL was observed. Each mouse was measured 3 times, with a 10-minute interval between each measurement, and the average value was calculated.

#### 2.4.6. Spleen and Thymus Indexes

In euthanized mice, the thymus and spleen were stripped, weighed, and the indices of the thymus and spleen were calculated. The thymus index = mass of thymus/mice body weight, and the spleen index = mass of spleen/mice body weight.

#### 2.4.7. Analysis of Plasma *β*-END, CRF, and IL-1*β* and in the Tumor and Surrounding Tissues by ELISA

Blood was harvested from the eyeballs of six mice from each group and immediately placed in clean eppendorf (EP) tubes with heparin, centrifuged for 10 minutes at 4°C, and the supernatant plasma was transferred to clean EP tubes and stored at −80°C for analysis. The right paw was depilated by 8% sodium sulfide solution, and the tumor and its surrounding tissues were obtained by deboning. A weight/volume ratio of 1 : 9 plus NS which was 10% of the homogenate (under low temperature) was centrifuged (3000 rpm/min) for 15 minutes at 4°C, and the superstratum plasma was transferred to clean EP tubes and stored at −80°C for analysis by ELISA according to the determination of *β*-END in the mouse kit.

#### 2.4.8. Immunohistochemical Analysis of *β*-END, POMC, and *μ*-OR in Tumor and Surrounding Tissues

The remaining six mice in each group were sacrificed to obtain the right hind paw. The paw was depilated by 8% sodium sulfide solution, fixed by 10% formaldehyde solution for 24 h, and decalcification by 30% acetic acid solution for 10 days. The tissue was then placed in 70% ethanol solution and paraffin-embedded to obtain immunohistochemistry slices. The streptomycinavidin-peroxidase assay was adopted for immunohistochemistry according to the appropriate kit. Positive results were observed under optical microscopy where hyalomitome and the cell membrane appeared brown (*β*-END or *μ*-OR).

### 2.5. Statistical Processing

All quantitative data derived from this study were analyzed statistically. The results were expressed as mean ± standard deviation (SD). Database was set up with SPSS 13.0 software package (SPSS Inc., Chicago, IL, USA). Differences among groups were analyzed by one-way analysis of variance (ANOVA). If the variance was regular, the *Q* test or Bonferroni test was used, and if the variance was irregular, the Tamhane method was used as a two-sided test. Resulting *P* values less than 0.05 were regarded as statistically significant.

## 3. Results

### 3.1. Establishment of Mouse Hind Paw Cancer Pain Model

**Figure 1:**
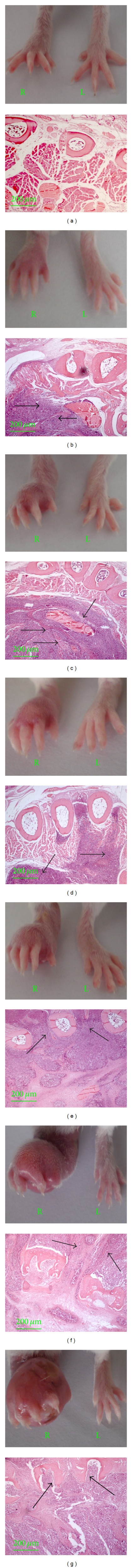
The representative morphological photographs of the mouse right and left paws and representative photomicrographs of mice right paws with hematoxylin and eosin on different days after injection of the cancer cell line H22. Upper images: representative morphological photographs of the mouse right and left paws; lower images: representative photomicrographs of mice right paws with hematoxylin and eosin, which were found to have a large number of cancer cells (labeled with blue arrowheads) on the 4th day after inoculation. There was a tendency for bone invasion which began on the 10th day, and bone tissue was destroyed in the first 20 days and was further damaged on the 30th day. (a) 0 day; (b) 4 days; (c) 6 days; (d) 8 days; (e) 10 days; (f) 20 days; (g) 30 days.

Figure 1 showed that the mouse paw cancer pain model was successfully established. Compared with the left paw (injected with normal saline), the volume of the right hind paw injected with H22 hepatoma cells gradually increased on visual inspection with the passage of time. The right paw was found to have a large number of cancer cells (labeled with blue arrowheads) on the 4th day after inoculation shown by hematoxylin and eosin. There was a tendency for bone invasion in the right hind paw, which began on the 10th day after inoculation, and bone tissue was destroyed in the first 20 days and was further damaged on the 30th day.

### 3.2. Effect of Cinobufagin on Thermal Hyperalgesia

**Figure 2:**
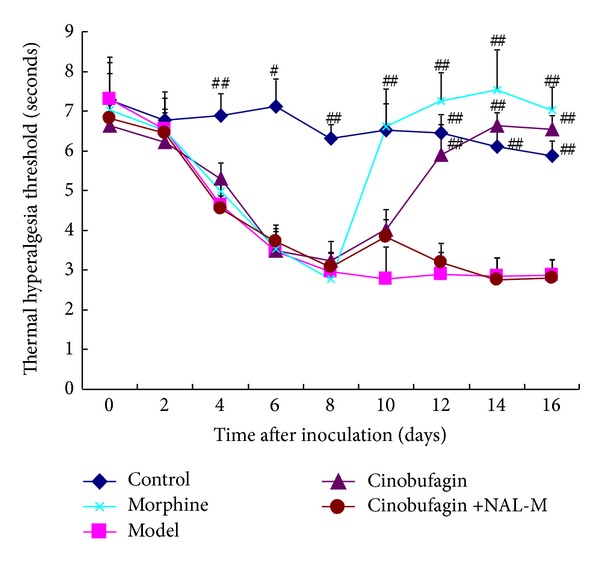
Effect of cinobufagin on thermal hyperalgesia at different times. Cinobufagin significantly increased the pain threshold on the 4th day, which increased gradually up to the 6th day, which was blocked by the peripheral opioid receptor antagonist NAL-M. Data are shown as the mean ± SD (*n* = 12). ^#^ *P* < 0.05, ^##^ *P* < 0.01 compared to model group.

The threshold of thermal pain of mice in the control group was 7 seconds, which was much higher (*P* < 0.01) than that following injection of H22 hepatoma cells (3 seconds) on the 8th day. The homologous drugs were administered from the 9th day, and morphine worked faster than the other treatments and immediately increased the pain threshold to normal. Cinobufagin also significantly increased the pain threshold on the 4th day, which increased gradually up to the 6th day. The effect of cinobufagin on thermal hyperalgesia was blocked by the peripheral opioid receptor antagonist NAL-M (Figure 2).

### 3.3. Effect of Cinobufagin on Mechanical Hyperalgesia

**Figure 3:**
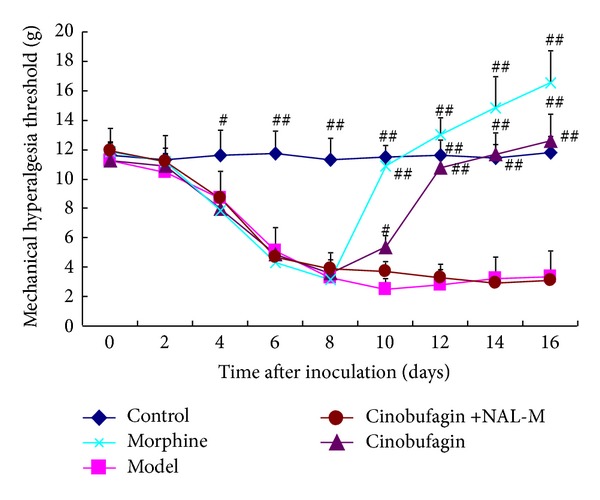
Effect of cinobufagin on mechanical hyperalgesia at different times. Cinobufagin significantly increased the pain threshold on the 4th day, which increased gradually up to the 8th day, which was blocked by the peripheral opioid receptor antagonist NAL-M. Data are shown as the mean ± SD (*n* = 12). ^#^ *P* < 0.05, ^##^ *P* < 0.01 compared to model group.

The threshold of mechanical pain in mice in the control group was 11.5 g, which was much higher than that following injection of H22 hepatoma cells (4.0 g) on the 8th day (*P* < 0.01). The homologous drugs were administered from the 9th day, and morphine worked immediately, increasing the pain threshold of mice to normal (*P* < 0.01). Cinobufagin also significantly increased the pain threshold on the 4th day (*P* < 0.01), which increased gradually up to the 8th day (*P* < 0.01). Furthermore, the thermal hyperalgesia effect of cinobufagin was blocked by the peripheral opioid receptor antagonist NAL-M (Figure 3).

### 3.4. Effect of Cinobufagin on the Spleen and Thymus Index

The results showed that the spleen and thymus indices in model group were significantly reduced compared with the control group (*P* < 0.05 and *P* < 0.01, resp.). However, cinobufagin significantly increased the spleen and thymus indices compared with the model group (*P* < 0.05 and *P* < 0.01, resp.), and morphine showed little effect on these immune organs compared with the model group (Table 1).

### 3.5. Effect of Cinobufagin on the Expressions of *β*-END, CRF, and IL-1*β* in the Right Hind Paw Tumor and Adjacent Tissue

The expression of *β*-END in the plasma, tumor, and adjacent tissue in cancer pain model mice was much lower than that of the control mice (*P* < 0.05 and *P* < 0.01, resp.). Cinobufagin significantly enhanced the expression of *β*-END of the tumor and surrounding tissues compared with the model group (*P* < 0.05 and *P* < 0.01, resp.). However, the level of *β*-END in plasma showed little change following treatment with Cinobufagin (Table 2). The expression of CRF and IL-1*β* in the plasma, tumor, and adjacent tissue in cancer pain model mice was much higher than that in the control mice (*P* < 0.01); after treatment with Cinobufagin, the expression of CRF was significantly increased in the tumor and adjacent tissue relative to the model group (*P* < 0.01). However, the level of CRF in plasma showed little change following treatment with cinobufagin. In addition, the peripheral opioid receptor blocking pharmacon, NAL-M, had a little effect on cinobufagin in increasing the level of plasm CRF (Table 2).

### 3.6. Effect of Cinobufagin on the Protein Expressions of *β*-END, POMC, and *μ*-OR in the Right Hind Paw Tumor and Adjacent Tissue

**Figure 4:**
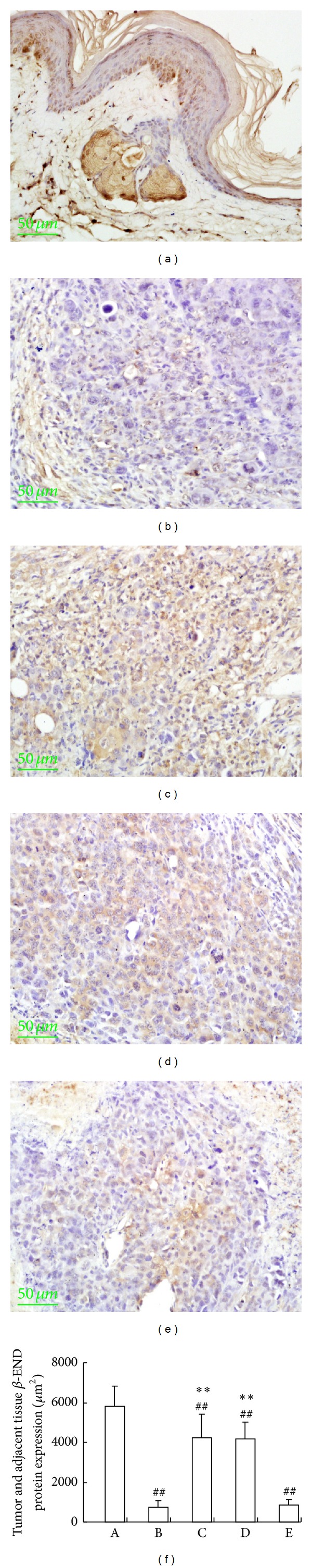
The representative photomicrographs of the tumor and adjacent tissues *β*-END immunohistochemistry (400x) and effect of Cinobufagin on the tumor and adjacent tissue *β*-END protein expressions in the cancer pain mice. (a) Control group; (b) model group; (c) cinobufagin group (Cinobufagin, 10 mL/kg/day, i.p.); (d) cinobufagin + NAL-M group (Cinobufagin, 10 mL/kg/day, NAL-M, 20 mg/kg/day i.p.); (e) morphine group (morphine, 8 mg/kg/day, i.p.). (f) Effect of Cinobufagin on the tumor and adjacent tissue *β*-END protein expression. Label with red arrowheads represents *β*-END positive expression. Data are shown as the mean ± SD (*n* = 6). ^#^ *P* < 0.05, ^##^ *P* < 0.01 compared to control group; *P < 0.05, **P < 0.01 compared to model group.

**Figure 5:**
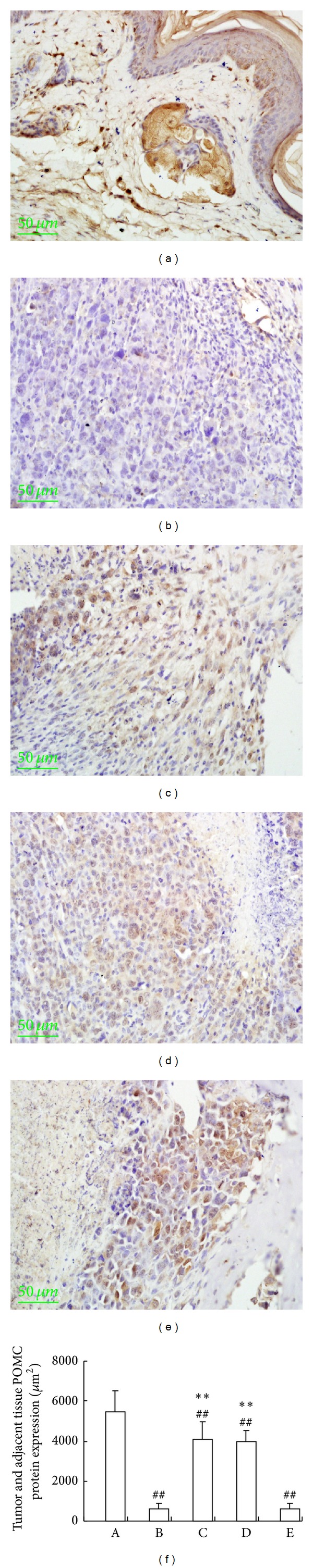
The representative photomicrographs of the tumor and adjacent tissues POMC immunohistochemistry (400x) and effect of Cinobufagin on the tumor and adjacent tissue POMC protein expressions in the cancer pain mice. (a) Control group; (b) model group; (c) cinobufagin group (cinobufagin, 10 mL/kg/day, i.p.); (d) cinobufagin + NAL-M group (cinobufagin, 10 mL/kg/day, NAL-M, 20 mg/kg/day i.p.); (e) morphine group (morphine, 8 mg/kg/day, i.p.). (f) Effect of cinobufagin on the tumor and adjacent tissue POMC protein expression. Label with red arrowheads represents POMC positive expression. Data are shown as the mean ± SD (*n* = 6). ^#^ *P* < 0.05, ^##^ *P* < 0.01 compared to control group; *P < 0.05, **P < 0.01 compared to model group.

**Figure 6:**
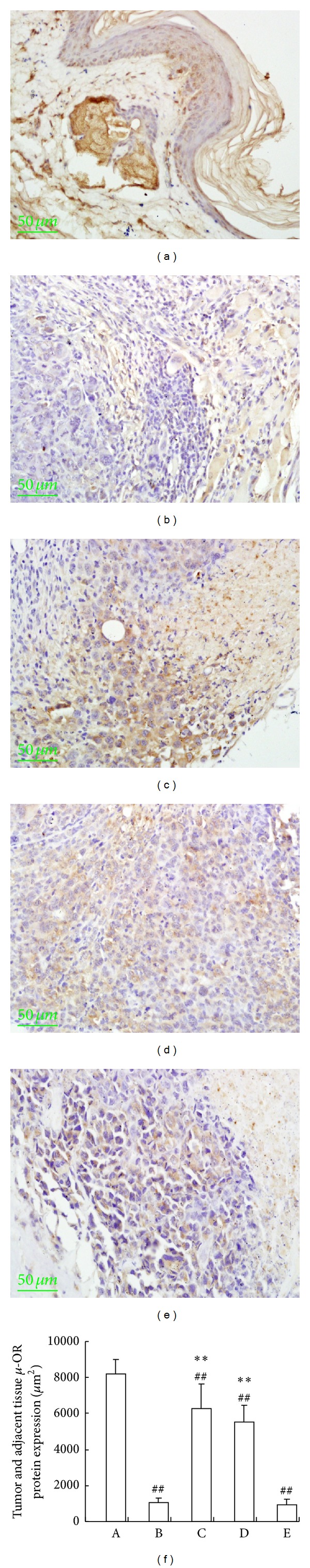
The representative photomicrographs of the tumor and adjacent tissues *μ*-OR immunohistochemistry (400x) and effect of Cinobufagin on the tumor and adjacent tissue *μ*-OR protein expressions in the cancer pain mice. (a) Control group; (b) model group; (c) cinobufagin group (cinobufagin, 10 mL/kg/day, i.p.); (d) cinobufagin + NAL-M group (cinobufagin, 10 mL/kg/day, NAL-M, 20 mg/kg/day i.p.); (e) morphine group (morphine, 8 mg/kg/day, i.p.). (f) Effect of cinobufagin on the tumor and adjacent tissue *μ*-OR protein expression. Label with red arrowheads represents *μ*-OR positive expression. Data are shown as the mean ± SD (*n* = 6). ^#^ *P* < 0.05, ^##^ *P* < 0.01 compared to control group; *P < 0.05, **P < 0.01 compared to model group.

In the control group, the results of immunohistochemistry showed that there was significant expression of *β*-END, POMC, and *μ*-OR and widespread distribution of positive brown-staining was observed in many hyalomitome (or cell membrane). However, there were few positive colored areas and fewer buff-colored cells in the model group, which meant that the expression of these cells in cancer pain mice was low. Cinobufagin markedly increased the number of cells which expressed *β*-END and the number of positive colored areas was much higher than that of cancer pain model mice. In contrast, morphine showed little influence on the expression of *β*-END, POMC, and *μ*-OR (Figures 4, 5, and 6).

## 4. Discussion

### 4.1. Establishment of the Hind Paw Cancer Pain Model

Mechanism elucidation and new therapies in cancer pain research have been impeded due to the lack of suitable experimental animal models of cancer pain. In 2001, Wacnik and his colleagues [19] established animal models of cancer pain by the transplantation of melanoma to different areas of the hind limbs. Following this, several laboratories used different methods to simulate cancer patients and established a variety of different animal models of tumor-induced pain [20]. Subsequently, Medhurst et al. [21–24] successfully established a rat model of bone cancer pain by inoculating MRMT-1 rat breast cancer cells into the tibia of Sprague-Dawley rats. The model showed hind limb weight reduction, mechanical hyperalgesia and allodynia, and other pain-related behavior. At the same time, many mouse models of cancer pain were established, including a bone cancer pain model in mice tibia, a femur cancer pain model, and heel pain models, and these models have made a significant contribution to elucidating the mechanisms of cancer pain and in the search for new drugs to treat cancer pain. However, most of the pain models are currently obtained by surgery, which causes pain and related injuries to the body and does not match clinical cancer pain.

In contrast, the experimental method by Lee et al. [25] in the HCa-1 cancer pain model in C3H/HeJ mice is simple and shows not only thermal hyperalgesia and mechanical hyperalgesia but also a “mirror pain” phenomenon. This pain model is more successful, causes less damage to the body, and can be used as an ideal model for the identification of drug treatment for cancer pain. Based on this model, the experimental H22 cancer pain model was established in this study. Cancer cell infiltration into the bone of the right hind paw was observed, and the results indicated that the pain model was successful, caused less damage to the body, and can be used as an ideal model to identify drugs for the treatment of cancer pain.

### 4.2. Evaluation of a Mouse Model of Bone Cancer Pain and Effect of Cinobufagin

In humans, tumors cause damage and a continuous dull pain. Due to aggravation of bone destruction, pain increases gradually and movement or touch can trigger acute pain. Based on this, the most important factor of an experimental animal model is that pain behavior should be similar to the human disease course. Its occurrence and degree are positively related to bone destruction and osteolytic activity [26]. In our present study, the animal model of cancer pain in this study was similar to the clinical manifestations of pain; before administration of the drugs, the threshold of mechanical pain was 11.5 g and the threshold of thermal pain was 7 seconds; on the fourth day after inoculation, behavioral signs of mechanical pain and radiant heat hyperalgesia were observed, respectively. The threshold of mechanical pain gradually worsened and dropped to 4.0 g on the 8th day, and the threshold of thermal pain decreased gradually to 3 seconds on the 8th day. “Mirror-image” pain developed in these animals from the 12th day, and significant mechanical and heat hyperalgesia lasted for 18 days. The ache in the hind paw injected with cancer cells at two days after administration was due to significant swelling compared to the normal rear paw (*P* < 0.05), and the ache gradually increased. The aching paw was found to have a large number of cancer cells on the 4th day after inoculation by hematoxylin and eosin. There was a tendency for bone invasion in the aching hind paw which began on the 10th day after inoculation, and the bone tissue was destroyed within the first 20 days, with further damage observed on the 30th day.

Administration of morphine from the 9th day had a rapid effect compared to the model group and increased the pain threshold of mice to normal levels. Cinobufagin significantly increased the threshold of thermal pain and mechanical pain on the 4th day after injection. Moreover, cinobufagin gradually induced a plateau in the threshold of mechanical pain on the 6th day and the thermal pain threshold plateaued on the 8th day after administration. The peripheral opioid receptor antagonist, NAL-M, eliminated the thermal hyperalgesia effect of cinobufagin.

### 4.3. Mechanism of Action of Cinobufagin in the Treatment of Cancer Pain

Recently, clinical researches have indicated that cinobufagin showed efficacy in raising the pain threshold, decreasing the extent of harmful reactions, relieving pain by changing the mental environment, and having the advantages of less toxicity and no addiction or withdrawal disorders. However, the mechanism of cinobufagin attenuating carcinoma pain is unclear. In this study, the effects of cinobufagin on the symptoms of cancer pain in mice, their pain threshold, and indices of immune organs were investigated, following the establishment of a mouse model of hind paw cancer pain. Results showed that cinobufagin could improve the symptoms of cancer pain mice and raise their pain threshold, whose mechanism may involve increasing content of *β*-END in the hind paw tumor and its surrounding tissue. The results also showed that its precursor protein, POMC, was substantially expressed, illustrating that cinobufagin markedly improved its synthesis by increasing the expression of POMC.

Considering other results, we could assume the process as follows: cinobufagin enhances the expression of POMC to increase the synthesis and release of *β*-END, raise the levels of CRF to boost the expression of *μ*-opioid receptor, and ultimately to increase the opportunity of *β*-END binding to the *μ*-opioid receptor to play an important role in peripheral analgesia.

## 5. Conclusion

In this study, Cinobufagin is better than morphine in the treatment of cancer pain. Although morphine works immediately, cinobufagin has an effect on peripheral opioid receptors, has no side effects such as addiction like morphine and other opioids, and can be used as a substitute for morphine in the treatment of patients with cancer pain. Until now, there has been little information on the mechanism underlying cancer pain. Our study provides a new method for investigating the mechanism and clinical treatment of cancer pain. The action mechanism of cinobufagin for the treatment of mouse hind paw cancer pain was mainly related to improving the level of peripheral *β*-END and the role of peripheral opioid receptors; however, the synthesis of *β*-END is complex [27], and the mechanism by which signaling pathways improve the synthesis and release of *β*-END is unclear. What types of immune cells have high expression of POMC, which cytokines are involved, and the influence of the peripheral immune-induced analgesia mechanism all require further study. The analgesic effects of cinobufagin on other types of cancer pain models and clarification of its specific molecular mechanism on enhancing the level of *β*-END will provide a more solid theoretical basis for the wide clinical use of cinobufagin.

*Statements.* Cinobufagin had been widely used in the treatment of carcinoma and played an important role in the relief of cancer pain. In this study the pain behavior index, immune function of the model, synthesis, and release of *β*-END in tumors and surrounding tissues and the expression of *μ*-ORs were investigated to identify the peripheral mechanisms of cinobufagin in alleviating cancer pain. 

## Figures and Tables

**Table 1:** Effect of cinobufagin on spleen and thymus indexes in the experimental group mice.

Group	Spleen index (mg/g)	Thymus index (mg/g)
Control	4.72 ± 0.88	3.53 ± 0.66
Model	3.56 ± 0.48^#^	2.80 ± 0.56^#^
Cinobufagin	5.67 ± 0.90^#∗∗^	4.10 ± 0.75**
Cinobufagin + NAL-M	4.86 ± 1.07*	3.42 ± 0.50*
Morphine	4.07 ± 0.76	299 ± 0.58

Control: control group; model: model group; cinobufagin group: cinobufagin, 10 mL/kg/day; cinobufagin + NAL-M group: cinobufagin, 10 mL/kg/day, NAL-M, 20 mg/kg/day; E; morphine group (morphine, 8 mg/kg/day, i.p.). Data are shown as the mean ± SD (*n* = 12). ^#^ *P* < 0.05, ^##^ *P* < 0.01 compared to control group; *P < 0.05, **P < 0.01 compared to model group.

**Table 2:** Effects of cinobufagin on homogenate and plasma *β*-END, CRF, and IL-1*β* in the experimental group mice.

Group	*β*-END (ng/L)		CRF (ng/L)		IL-1*β* (ng/L)	
	Homogenate	Plasma	Homogenate	Plasma	Homogenate	Plasma
Control	188.04 ± 39.43	154.52 ± 22.44	69.93 ± 4.31	74.67 ± 7.69	55.39 ± 13.45	62.14 ± 9.42
Model	54.75 ± 14.41^##^	72.54 ± 23.85^##^	143.24 ± 25.09^##^	161.35 ± 12.24^##^	147.90 ± 26.08^##^	148.30 ± 18.57^##^
Cinobufagin	166.16 ± 43.01^##∗∗^	64.02 ± 9.96^##^	310.78 ± 65.66^##∗∗^	131.88 ± 10.21^##^	157.51 ± 20.12^##^	161.19 ± 26.49^##^
Cinobufagin + NAL-M	159.18 ± 29.14^#∗∗^	75.14 ± 13.92^##^	295.88 ± 57.29^##∗∗^	138.68 ± 13.58^##^	165.33 ± 19.42^##^	149.99 ± 9.26^##^
Morphine	68.58 ± 17.79^##^	71.38 ± 8.79^##^	138.03 ± 17.79^##^	140.59 ± 44.94^##^	160.64 ± 19.33^##^	139.47 ± 15.54^##^

Control: control group; model: model group; cinobufagin group: cinobufagin, 10 mL/kg/day; cinobufagin + NAL-M group: cinobufagin, 10 mL/kg/day, NAL-M, 20 mg/kg/day; E; Morphine group: morphine, 8 mg/kg/day, i.p. Data are shown as the mean ± SD (*n* = 6). ^#^ *P* < 0.05, ^##^ *P* < 0.01 compared to control group; *P < 0.05, **P < 0.01 compared to model group.

## Abbreviations

ACTH: Adrenocorticotropic hormone
*α*-MSH: Alpha melanocyte stimulating hormone
*β*-END: Beta endorphin
CRF: Corticotropin releasing factor
ELISA: Enzyme linked immunosorbent assay
IL-1*β*: Interleukin-1 beta
*μ*-OR: Mu-opioid receptor
NAL-M: Naloxone methiodide
NS: Normal saline
POMC: Proopiomelanocortin
PWL: Paw withdrawal latency.
